# Artificial Versus Human Intelligence in the Diagnostic Approach of Ophthalmic Case Scenarios: A Qualitative Evaluation of Performance and Consistency

**DOI:** 10.7759/cureus.62471

**Published:** 2024-06-16

**Authors:** Achilleas Mandalos, Dimitrios Tsouris

**Affiliations:** 1 Ophthalmology, General Hospital of Karditsa, Karditsa, GRC; 2 Ophthalmology, General University Hospital of Larissa, Larissa, GRC

**Keywords:** challenging ophthalmic cases, diagnostic approach, google gemini, bing copilot, chatgpt, artificial intelligence

## Abstract

Purpose: To evaluate the efficiency of three artificial intelligence (AI) chatbots (ChatGPT-3.5 (OpenAI, San Francisco, California, United States), Bing Copilot (Microsoft Corporation, Redmond, Washington, United States), Google Gemini (Google LLC, Mountain View, California, United States)) in assisting the ophthalmologist in the diagnostic approach and management of challenging ophthalmic cases and compare their performance with that of a practicing human ophthalmic specialist. The secondary aim was to assess the short- and medium-term consistency of ChatGPT’s responses.

Methods: Eleven ophthalmic case scenarios of variable complexity were presented to the AI chatbots and to an ophthalmic specialist in a stepwise fashion. Advice regarding the initial differential diagnosis, the final diagnosis, further investigation, and management was asked for. One month later, the same process was repeated twice on the same day for ChatGPT only.

Results: The individual diagnostic performance of all three AI chatbots was inferior to that of the ophthalmic specialist; however, they provided useful complementary input in the diagnostic algorithm. This was especially true for ChatGPT and Bing Copilot. ChatGPT exhibited reasonable short- and medium-term consistency, with the mean Jaccard similarity coefficient of responses varying between 0.58 and 0.76.

Conclusion: AI chatbots may act as useful assisting tools in the diagnosis and management of challenging ophthalmic cases; however, their responses should be scrutinized for potential inaccuracies, and by no means can they replace consultation with an ophthalmic specialist.

## Introduction

Despite being developed as conversational artificial intelligence (AI) systems, large language model (LLM)-based chatbots are currently the focus of intense interest in the field of medicine, particularly in ophthalmology. For example, Chat Generative Pre-trained Transformer (ChatGPT) (OpenAI, San Francisco, California, United States), released in late 2022, has already shown remarkable ability in providing general information and advice for glaucoma [1] and age-related macular degeneration patients [2], answering ophthalmology StatPearls questions [3], and even helping triage ophthalmic emergency cases [4].

AI chatbots have been able to diagnose ophthalmic cases when presented with the full case description, for example, cases extracted from publicly available online clinical databases [5]. This is relatively easy when the description includes all relevant data and certain keywords of the eye condition in question. However, in real life, it rarely happens that the ophthalmologist has the full clinical and laboratory data available, and most often a stepwise approach is followed in order to reach the diagnosis (i.e. first listening to the patient’s description of symptoms and forming an initial differential diagnosis list, then proceeding to clinical examination and refining our differential diagnosis, then deciding which laboratory and/or imaging tests are needed to narrow our differential diagnosis list further, etc.).

Thus, we conducted this qualitative descriptive study with the aim to assess and comparatively evaluate the efficiency of three of the most widely used AI chatbots (ChatGPT-3.5, Bing Copilot (Microsoft Corporation, Redmond, Washington, United States), Google Gemini (Google LLC, Mountain View, California, United States)) in assisting the ophthalmologist in this stepwise process to diagnose and manage certain, sometimes challenging, ophthalmic cases and compare their responses with those of a human ophthalmic specialist. Especially with regard to ChatGPT, we were also interested in assessing its short-term (same day) and medium-term (one month apart) consistency in responses as well as its ability to improve its diagnostic performance over time.

## Materials and methods

This study was conducted between November 2023 and February 2024 at the General Hospital of Karditsa, Karditsa, Greece. No identifiable personal data were included in the case scenarios and thus no ethics approval was necessary. Of note, at the time of conducting our study, Google Gemini was still known as Google Bard.

Eleven ophthalmic case scenarios (either factual or fictional) of variable diagnostic difficulty and encompassing various ophthalmic subspecialties were presented to all three AI chatbots and a human ophthalmologist with more than 10 years of post-fellowship clinical experience, who was blind to the diagnoses following the methodology described as follows: (i) Patients' complaints were presented and an initial differential diagnosis was requested and (ii) Clinical examination and relevant laboratory findings were presented and a refined diagnosis was requested. If the diagnosis provided by the chatbot was incorrect, further suggestions were offered to rectify it. If the correct diagnosis was provided by the chatbot, depending on the case, advice on further investigation and management was requested (if not already offered by the chatbot).

A conversational language was used in the interaction with the chatbots, trying to mimic the interaction between humans. To make the process more challenging for both the chatbots and the human ophthalmologist, not all typical symptoms and/or signs of the condition in question were presented in every case and even confounding factors or equivocal findings were introduced in certain cases. We wanted to mimic real life and evaluate the ability of AI to contribute to the diagnostic thought process based on available and sometimes confounding, rather than complete and fully fitting, clinical data.

The above process was repeated one month later for ChatGPT only, using as input the exact same phrasing as the first time. If the correct diagnosis was offered at an earlier stage, no further prompts were given. The exact same process was repeated once again on the same day (i.e. at the one-month time point) to assess the short-term consistency of ChatGPT responses.

The medium-term consistency of ChatGPT responses was evaluated by critically comparing its initial responses to those given one month later. We also evaluated whether it was able to reach the correct diagnosis earlier on that second occasion. Where ChatGPT’s responses could be described as items (i.e. item list of initial differential diagnoses and diagnostic workup), the similarity of responses was assessed using the Jaccard similarity coefficient. Otherwise, due to the descriptive nature of the study and the small number of case scenarios involved no formal statistical analysis was performed.

## Results

Details of the case scenarios and diagnoses are presented in Table 1

**Table 1:** Case scenarios included in the study (diagnosis and sequence of description/questions used as input to chatbots). Examples of further prompts given to chatbots in case of erroneous response are presented in italics. IOP: intraocular pressure; AC: anterior chamber; PMH: previous medical history; MRI: magnetic resonance imaging; ACE: angiotensin converting enzyme; ESR: erythrocyte sedimentation rate; CRP: C-reactive protein; CBC: complete blood count; VA: visual acuity; CRVO: central retinal vein occlusion; AMD: age-related macular degeneration

Case	Diagnosis	Description/Questions used as input to chatbots
1	Aqueous misdirection after trabeculectomy	“Man who underwent trabeculectomy 3 days ago calls to say that he has sudden painful loss of vision. Differential diagnosis?”
		“On examination IOP=50mmHg, almost flat AC. Fundus looks ok. Diagnosis?”
		“The bleb is flat and the IOP is definitely high, so no hypotony. Also macula was ok on fundoscopy, so no maculopathy. Any other thoughts?”
2	Microischemic (diabetes-related) sixth nerve palsy	“A 75-year-old man complains of sudden onset horizontal diplopia. No pain. Differential diagnosis?”
		“On examination patient has left eye esotropia with limitation of left eye abduction. PMH: diabetes. Diagnosis?”
		“What MRI scans and what blood tests should I order for this patient?”
3	Anterior uveitis secondary to sarcoidosis	“47 year old female complains of red right eye with blurry vision and photophobia of a few days duration. Differential diagnosis?”
		“On examination conjunctiva is red, there is limbal injection, cornea is clear but with a few inferior endothelial precipitates, AC deep with cells ++. Diagnosis?”
		“Any treatment regimen you can suggest?”
		“She says this has happened twice in the past. What uveitis laboratory workup should I order?”
		“Patient says she has had a cough for the last few months Could this be related to her uveitis and if yes, what additional tests should I order?”
		“Labs have come back: ACE is high and also the chest X-ray shows hilar lymphadenopathy. Diagnosis?”
4	Central retinal artery occlusion (CRAO) associated with giant cell arteritis (GCA)	“70-year-old woman complains of sudden painless loss of vision, vision= hand movement. Differential diagnosis?”
		“Fundoscopy showed CRAO. What lab workup should I order?”
		“ESR is 30mm/hour, CRP 4 mg/L, Glucose 100mg/dl. Lipids and CBC normal. Most probable diagnosis?”
5	Charles Bonnet syndrome	“80-year-old man with end-stage glaucoma and visual acuity of light perception in right eye and hand movement in left eye complains of seeing funny objects and animals moving about in his room at bedtime. Differential diagnosis?”
6	Central serous retinochoroidopathy (CSR)	“43-year=old man, lorry driver, complains of blurry vision mostly left eye with difficulty focusing after long stints of driving in the past week and lack of sleep. He says he is myopic -1.0 D in both eyes. On VA test, he can see 10/10 Snellen with -1.0 D in right eye, but in left eye he reaches 10/10 Snellen with plano refraction (ie. shift towards emmetropia). Any ideas about the diagnosis?”
		“Amsler chart normal in right eye, but blurry centrally in left eye. Patient says he has been under a lot of stress recently. On fundoscopy left eye macula shows well-circumscribed area of edema-elevation. Diagnosis?”
		“Not diabetic, definitely no CRVO, no uveitis evident, no AMD (remember he is only 43 years old), no epiretinal membrane on fundoscopy. Any other ideas?”
		“Recall that he is young (43 years old), stressed-type, with unilateral well-circumscribed macula elevation and hyperopization (his myopia has reduced from -1.0 D to plano refraction). Any ideas?”
		“What is the current evidence-based management of this condition?”
7	Idiopathic intracranial hypertension (IIH)	“29-year-old woman complains of blurry vision since last 3 days, headaches worse in the morning. VA 7/10 Snellen both eyes, eyes white-quiet, but on fundoscopy indistinct papillary margin in both eyes. Differential diagnosis? What questions would you further ask the patient to clarify diagnosis?”
		“Headaches started 2 weeks ago, in the morning and worse when bending down, come and go. Nausea yes, but no vomiting. No other neurological symptoms. On diet pills (she is overweight). No previous history of hypertension, today blood pressure 120/80 mmHg. Diagnosis?”
8	Acute third nerve palsy due to posterior communicating artery (PCA) aneurysm	“44-year-old man complains of sudden-onset diplopia and headache. On examination left eye is squinting outwards and downwards, there is limitation of adduction. Also left eye lid ptosis. Left eye pupil is larger. Diagnosis and further investigation and management?”
9	Orbital pseudotumor	“38-year-old man complains of sudden-onset painful proptosis of right eye with diplopia. On examination there is right eye proptosis with limitation of eye movement especially abduction. Also pain around the eye. No known thyroid disease. Differential diagnosis?”
		“Based on clinical examination and previous history I suspect he has orbital pseudotumor/Tolosa Hunt syndrome. What investigation should I do to confirm?”
10	Acute angle closure glaucoma attack (AACG)	“79-year-old female complains of acute pain around the right eye, blurry vision and nausea for the last few hours. In the last few days occasionally haloes around lights lasting for a few minutes. Diagnosis?”
		“Ophthalmologist checked IOP= 55mmHg, hazy cornea, fixed mid-dilated pupil, not possible to do gonioscopy due to hazy cornea. Shallow anterior chamber, Van Herrick 1:4. Diagnosis?”
		“Can you give me a management plan for AACG?”
11	Marginal keratitis	“27-year-old contact lens wearer complains of red right eye and pain. Differential diagnosis?”
		“On examination, right eye is conjunctival and episcleral injection mostly marked near the site of a small peripheral corneal stromal infiltration with bridging vessels from limbus. No corneal ulcer. Also marked blepharitis. Diagnosis?”
		“But there is no ulceration and patient is systemically healthy. Any other diagnosis?”
		“How to manage this condition?”

Examples of human and AI responses are presented in Tables 2, 3.

**Table 2:** Human and AI responses in Case 1 (aqueous misdirection after trabeculectomy). IOP: intraocular pressure; AC: anterior chamber; CRVO: central retinal vein occlusion; AI: artificial intelligence

Human	ChatGPT	ChatGPT one month later	Bing Copilot	Google Bard/Gemini
“Man who underwent trabeculectomy three days ago calls to say that he has sudden painful loss of vision. Differential diagnosis?				
Bleb-related infection; Aqueous misdirection; Choroidal effusion (“kissing choroidals”)	Hypotony; Choroidal effusion; Endophthalmitis; Hyphema; Corneal edema; Cystoid macular edema; Retinal detachment; Suprachoroidal hemorrhage	Infection; Endophthalmitis; Hemorrhage; Choroidal effusion; Hyphema; Corneal edema; Malignant glaucoma; Detached retina	Postoperative complications (infection, inflammation, or hemorrhage); Increased or decreased intraocular pressure; Cystoid macular edema; Bleb-related infections/endophthalmitis; Corneal edema or decompensation	Delayed suprachoroidal hemorrhage; Choroidal effusion; Corneal infection; Hyphema; Hypotony; Corneal graft rejection
“On examination IOP=50mmHg, almost flat AC. Fundus looks ok. Diagnosis?”				
Aqueous misdirection	Hypotony Maculopathy	Malignant glaucoma	Aqueous misdirection	Retinal detachment; Optic nerve ischemia; Central retinal vein occlusion; Acute angle-closure glaucoma; Ciliary body ischemia
	“The bleb is flat and the IOP is definitely high, so no hypotony. Also, macula was ok on fundoscopy, so no maculopathy. Any other thoughts?”			“There is no optic nerve ischemia, no CRVO, no retinal detachment. However the angle does look closed and AC is flat (iris-lens diaphragm in touch with cornea), bleb is flat (so no filtration). Diagnosis?”
	Malignant glaucoma			Malignant glaucoma; Cicatricial membrane formation; Hyphema with angle closure; Uveal effusion syndrome; Other less common causes (certain medications, trauma, or underlying anatomical abnormalities)

**Table 3:** Human and AI responses regarding diagnostic workup in case 3 (anterior uveitis secondary to sarcoidosis). ACE: angiotensin converting enzyme; FBC: full blood count; ESR: erythrocyte sedimentation rate; CRP: C-reactive protein; ANA: anti-nuclear antibodies; RF: rheumatoid factor; HLA: human leukocyte antigen; VDRL: venereal disease research laboratory; RPR: rapid plasma reagin; MRI: magnetic resonance imaging; CT: computed tomography; TBC: tuberculosis; RA: rheumatoid arthritis; SLE: systemic lupus erythematosus; AI: artificial intelligence

Human	ChatGPT	ChatGPT one month later	Bing Copilot	Google Bard/Gemini
“She says this has happened twice in the past. What uveitis laboratory workup should I order?”				
Chest X ray; Serum ACE; Syphilis serology; FBC; ESR; CRP	FBC; ESR; CRP; ANA; RF; HLA-B27; Syphilis serology; Lyme disease serology; Tuberculosis testing; ACE; Quantiferon Gold Test	FBC; ESR; CRP; ANA; RF; anti-cyclic citrullinated peptide; HLA-B27; Syphilis and Tuberculosis testing (e.g., VDRL or RPR, interferon-gamma release assays or tuberculin skin test); Lyme disease testing; Imaging studies, such as chest X-rays or MRI scans	FBC; ESR; CRP; ACE in serum; Syphilis serology; HLA-B27; Chest X-ray or CT	I am strictly prohibited from recommending specific diagnostic tests or treatment plans.
“Patient says she has had a cough for the last few months Could this be related to her uveitis and if yes, what additional tests should I order?”				
Quantiferon Gold test; Mantoux test; Biopsy of potential conjunctival lesions	Sarcoidosis (chest X-ray); Tuberculosis (chest X-ray, sputum analysis, TBC skin test or Quantiferon Gold blood test); Connective Tissue Disorders such as RA or SLE (ANA, RF); Pulmonary Function Tests; High-Resolution Computed Tomography of the Chest; Sputum Analysis; Consultation with a Pulmonologist	Chest X-ray or Chest CT Scan; Pulmonary Function Tests; Infectious Disease Testing: sputum cultures, TBC skin tests, or interferon-gamma release assays; Autoimmune and rheumatologic Tests; Inflammatory markers: ESR, CRP	Yes (eg, sarcoidosis, TBC): Chest X-ray or CT scan; Tuberculin skin test or Interferon Gamma Release Assay; Serum ACE; Serum Lysozyme; Pulmonary function tests	I'm still unable to offer any specific medical advice or recommend diagnostic tests.

When asked for an initial differential diagnosis, both human and AI participants provided a reasonable average number of provisional diagnoses, albeit with a wide variation in the number of diagnoses, depending on the scenario presented (Table 4). Remarkably, only the ophthalmologist included the correct diagnosis in the initial list in all cases. In many cases the chatbots proposed additional appropriate potential diagnoses; however, they also occasionally included inappropriate or highly improbable diagnoses (for example, ChatGPT included retinal detachment in the differential diagnosis of the sudden painful loss of vision after trabeculectomy).

**Table 4:** Descriptive analysis of initial differential diagnosis. * Gemini refused to give any DDx on three occasions; on another three occasions, we needed to insist on getting a DDx. DDx: differential diagnosis; Dx: diagnosis; N/A: not applicable

	Initial DDx items: Median (range)	Number of scenarios with correct Dx included in the initial DDx	Number of scenarios with inappropriate/highly improbable Dx included in initial DDx	Number of scenarios where AI chatbot gave additional appropriate DDx which was not offered by human	Additional appropriate DDx items offered by AI chatbot: Median (range)
Human	7 (1-14)	11	0	N/A	N/A
ChatGPT	7 (1-10)	8	6	9	2 (1-7)
ChatGPT (1 month later)	7 (1-8)	9	6	7	3 (1-6)
Bing	4 (1-10)	7	5	5	1 (1-3)
Gemini	5 (0-10) *	3	4	5	2 (1-2)

Where confronted with the task of providing a definite diagnosis, the human ophthalmologist performed better than AI chatbots, missing only one diagnosis due to atypical clinical presentation and equivocal laboratory findings (Table 5). Cases 6 (CSR) and 11 (marginal keratitis) usually required further prompting and were not always correctly diagnosed by the chatbots.

**Table 5:** Descriptive analysis of final diagnosis (Dx) after presentation of clinical/laboratory findings. * Out of 9 scenarios where we asked for a final Dx (scenarios 1-4, 6-8, 10, 11). GCA: giant cell arteritis; CSR: central serous retinopathy; IIH: idiopathic intracranial hypertension; AACG: acute angle closure glaucoma

	Number of scenarios* correctly diagnosed	Comments
Human	8	In case 4 (GCA) Dx was missed because of equivocal lab findings and absence of typical systemic symptoms.
ChatGPT	7	In cases 1 (aqueous misdirection) and 6 (CSR) correct Dx was reached after further prompting.
ChatGPT (one month later)	7	In cases 6 (CSR) and 11 (marginal keratitis) correct Dx was reached after further prompting.
Bing	6	In case 4 (GCA) Dx was missed because of equivocal lab findings. In case 7 (IIH) partially correct Dx (“high intracranial pressure”) was provided. In case 11 (marginal keratitis) no correct Dx was offered even after prompting.
Gemini	4	In case 4 (GCA) Dx was missed because of equivocal lab findings. In cases 1 (aqueous misdirection), 6 (CSR), and 10 (AACG) correct Dx was reached after further prompting. In case 11 (marginal keratitis) no correct Dx was offered even after prompting.

Qualitative evaluation of AI chatbots per case scenario

Case 1 (Aqueous Misdirection After Trabeculectomy)

ChatGPT showed better diagnostic performance at one month. While the first time it needed further prompting to reach the correct diagnosis, in our second interaction, it included it in the initial list of potential diagnoses and was able to correctly diagnose the pathology upon presentation of clinical findings.

Case 2 (Diabetic Sixth Nerve Palsy)

Despite the microischemic nature of nerve palsy and the inappropriateness of any immediate diagnostic imaging in this case (as correctly identified by the human ophthalmologist), all three AI chatbots advised prompt brain MRI. In relation to blood tests, ChatGPT offered an appropriate laboratory workup similar to the one proposed by the human ophthalmologist, whereas Bing and Gemini responses were incomplete and contained unnecessary tests (eg. autoimmune tests or tests for syphilis, Lyme disease, and tuberculosis).

Case 3 (Anterior Uveitis Secondary to Sarcoidosis)

Apart from an initial differential diagnosis, Gemini refused to provide any further input in this case, repeatedly referring to its restrictions and prohibitions to provide medical advice. By contrast, both ChatGPT and Bing offered generally correct advice regarding treatment for anterior uveitis and complemented the laboratory workup proposed by the human ophthalmologist. Furthermore, both of them reached the correct systemic diagnosis (i.e. sarcoidosis) when presented with the laboratory findings.

Case 4 (Central Retinal Artery Occlusion (CRAO) Secondary to Giant Cell Arteritis (GCA))

In this case, all three AI chatbots provided a reasonable diagnostic workup for CRAO without major omissions, which complemented the already quite comprehensive plan proposed by the human ophthalmologist. With regard to the systemic diagnosis underlying CRAO, responses varied. ChatGPT gave a clear-cut diagnosis of GCA and proposed further management (ie. temporal artery biopsy and initiation of high-dose systemic steroids). Gemini acknowledged GCA as a possibility but stated that the C-reactive protein (CRP) value was indecisive at this point. By contrast, the human ophthalmologist and Bing offered non-arteritic central retinal artery ischemia as the final diagnosis. Bing justified its response by invoking the equivocal CRP value, while the human specialist added the absence of relevant GCA symptoms (e.g. temporal headache, jaw claudication) as additional factors affecting his diagnostic thinking. Interestingly, when we later returned to our discussion with Bing and added symptoms such as jaw claudication and shoulder pain to the case scenario, it changed its diagnosis to arteritic CRAO.

Case 5 (Charles Bonnet syndrome)

All three chatbots recognized the clinical entity of Charles Bonnet syndrome and included it in the differential diagnosis list.

Case 6 (Central Serous Retinochoroidopathy (CSR))

This case proved to be a tough diagnostic challenge for ChatGPT and Gemini, as they both needed further prompting to reach the correct diagnosis, while Bing provided the correct diagnosis as soon as the clinical findings were presented. By comparison, the human ophthalmologist was the only one to include CSR in the initial differential diagnosis and offered it as the final diagnosis after the presentation of clinical findings. When asked for evidence-based advice on treatment for CSR all three chatbots provided correct answers, although they did not always mention that acetazolamide and aldosterone antagonists are still off-label and do not form part of the standard treatment.

Case 7 (Idiopathic Intracranial Hypertension (IIH))

In general, the “questions to ask the patient” were common in the human and chatbots' responses (e.g. weight gain, medications, pregnancy, nausea-vomiting, changes in vision, diabetes, systemic hypertension). AI chatbots complemented the human ophthalmologist with some additional questions to ask the patient (e.g. onset, duration, and characteristics of headaches and their association with posture or activities). Regarding final diagnosis, IIH was offered as the most probable (but not exclusive) diagnosis by ChatGPT and Gemini, whereas Bing acknowledged high intracranial pressure as the cause of the patient's symptoms and clinical signs, but did not provide any specific diagnosis.

*Case 8 (Third Nerve Palsy Due to Posterior Communicating Artery (PCA)* *Aneurysm)*

AI chatbots offered correct advice regarding diagnostic workup (e.g. brain imaging, blood tests), but their investigation plan was not as comprehensive as that of the human ophthalmologist and, most importantly, they did not always recognize the urgency of the situation. In fact, Bing, but not Gemini, emphasized the need for urgent imaging, while ChatGPT did not advise urgent investigation until our second interaction one month later.

Case 9 (Orbital Pseudotumor)

Chatbots provided a reasonable, albeit incomplete, diagnostic workup plan which nevertheless complemented that which was proposed by the human ophthalmologist (e.g. orbital biopsy and thyroid function tests were not included in the ophthalmologist's response but they were advised by all three chatbots).

Case 10 (Acute Angle Closure Glaucoma Attack (AACG))

Of the three chatbots, ChatGPT provided the most comprehensive management plan including medication doses and timing, while the other two chatbots offered general advice regarding medical and surgical management. This was particularly true for Gemini, which clearly stated that it could only provide general information about potential treatment approaches. However, even ChatGPT was not so comprehensive in its response one month later. Of note, all three chatbots omitted the use of topical steroids. Upon further prompting, they acknowledged their omission and provided a rectified plan. Furthermore, they were able to engage in a conversation regarding the potential inappropriateness of using topical pilocarpine in the acute phase of ACG with a very high intraocular pressure (IOP) and a paralyzed iris sphincter muscle, providing justified arguments. However, it should also be noted that this may not always be the case, as the response of ChatGPT upon testing its short-term consistency at the one-month time point was deemed inaccurate (in our first interaction at one month, it acknowledged that pilocarpine may be ineffective at very high IOP, whereas in our second interaction on the same day, it insisted that pilocarpine may stimulate the paralyzed iris sphincter muscle and its effectiveness is only limited in case of complete angle closure or significant corneal haze).

Case 11 (Marginal Keratitis)

Of the chatbots, only ChatGPT was able to correctly diagnose this case but its performance at one month was weaker, as it required some prompting to reach the diagnosis. With regard to advice on management, ChatGPT provided the most comprehensive plan.

Consistency of ChatGPT responses

Consistency of ChatGPT responses one month apart is shown in Table 6. Jaccard similarity coefficient showed on average a fair overlap of items included in the initial differential diagnosis and diagnostic workup on these two separate occasions. With regard to final diagnosis, ChatGPT offered the correct diagnosis (either as stand-alone diagnosis or as part of further differential diagnosis) in all cases, both the first time and one month later. However, it occasionally needed further prompting in order to reach the correct diagnosis. Case 6 (CSR) turned out to be the most challenging case to diagnose, as it needed additional prompting at both times.

**Table 6:** Medium-term consistency of ChatGPT responses. * required further prompting; ** correct Dx offered as part of further DDx DDx: differential diagnosis; Dx: diagnosis; N/A: not applicable

	Jaccard similarity coefficient	
	Mean (SD)	Range
Initial DDx	0.58 (0.16)	0.33-0.8
Lab/imaging workup	0.66 (0.15)	0.5-0.9
Final Dx	Correct Dx (Yes / No)	
Case	1^st^ time	1 month later
1	Yes ^*^	Yes
2	Yes	Yes
3	Yes	Yes
4	Yes	Yes
5	N/A	N/A
6	Yes ^*^	Yes ^*^
7	Yes ^**^	Yes ^**^
8	Yes ^**^	Yes ^**^
9	N/A	N/A
10	Yes	Yes
11	Yes	Yes ^*^^/^^**^

Short-term consistency of ChatGPT responses (Table 7) as measured by Jaccard similarity coefficient seemed to be slightly better than medium-term consistency. However, concerning the final diagnosis, there were two occasions (case 6 and case 7) where although it offered the correct diagnosis the first time, upon repeating the process on the same day it failed to accurately diagnose the condition. For example, in case 7 (IIH), ChatGPT recognized the high intracranial pressure issue, but did not offer IIH as the potential cause.

**Table 7:** Short-term consistency of ChatGPT responses. * required further prompting; ** correct Dx offered as part of further DDx; *** recognized high intracranial pressure problem, but did not give the definite Dx (idiopathic intracranial hypertension) DDx: differential diagnosis; Dx: diagnosis; N/A: not applicable

	Jaccard similarity coefficient	
	Mean (SD)	Range
Initial DDx	0.76 (0.15)	0.5-1
Lab/imaging workup	0.66 (0.20)	0.5-1
Final Dx	Correct Dx (Yes / No)	
Case	1^st^ time	2^nd^ time
1	Yes	Yes
2	Yes	Yes
3	Yes	Yes
4	Yes	Yes
5	N/A	N/A
6	Yes ^*^	No
7	Yes ^**^	No ^***^
8	Yes ^**^	Yes ^**^
9	N/A	N/A
10	Yes	Yes
11	Yes ^*^^/ **^	Yes ^*^^/ **^

## Discussion

In the current study, AI chatbots, especially ChatGPT-3.5 and Bing Copilot, demonstrated remarkable ability as assisting tools in diagnosing and managing challenging ophthalmic cases of various subspecialties. Their individual performance was inferior to that of an ophthalmologist with more than 10 years of post-fellowship clinical experience, but still, they provided useful complementary input in the diagnostic thinking process. This is particularly impressive given that they have not been developed as medical diagnostic tools and they quite often make this clear to the reader before they proceed with their response to a medical query; Gemini, in particular, refused on some occasions to provide medical insight invoking its restrictions to serve as a medical tool. It is thus for this reason, as well as their occasional so-called “artificial hallucinations” [6], that their responses should be examined thoroughly for potential inaccuracies and errors and cross-checked with a specialist in case of doubt.

There is an emerging literature comparing AI chatbots, mainly ChatGPT, with humans as regards their diagnostic accuracy in ophthalmology. For example, ChatGPT-3.5 had similar or better accuracy than senior ophthalmology residents in diagnosing primary and secondary glaucoma cases retrieved from a public online database [7]. Similarly, ChatGPT-4 outperformed glaucoma specialists and was comparable with retina specialists in diagnostic and treatment accuracy of glaucoma and retina cases [8]. By contrast, ChatGPT exhibited reasonable but inferior diagnostic accuracy than human experts in cornea [9], uveitis [10,11], and neuro-ophthalmology [12] cases. Furthermore, in another study, performance of ChatGPT-3.5 in diagnosing hospitalized ophthalmic patients with various, sometimes complex, eye conditions was poorer than that of residents and attending ophthalmologists [13]. In our study we used the free-of-charge, readily accessible 3.5 version of ChatGPT and compared it against a quite experienced ophthalmologist. Given the superior performance of ChatGPT-4 compared to the 3.5 version it could be that the incorporation of this newest version in the diagnostic toolbox could potentially increase the diagnostic accuracy of ophthalmologists even more, but it should be emphasized that any chatbot may be used simply as an assistant, not as the definitive diagnostic tool.

When it comes to comparing different AI chatbots, current literature suggests better performance of ChatGPT than Google Bard/Gemini in triaging and diagnosing simulated ophthalmic patient complaints [14] or in providing an accurate and coherent surgical plan for glaucoma [15] and vitreoretinal [16] cases. Furthermore, ChatGPT-3.5 was found to be more accurate than Bing and Google Bard/Gemini in answering patients’ questions about age-related macular degeneration [2]. Moreover, in a study evaluating the performance of ChatGPT (versions 3.5 and 4) and Google Bard/Gemini in answering common inquiries regarding ocular symptoms, it was found that ChatGPT-4 outperformed ChatGPT-3.5 and Google Bard; however, all chatbots exhibited only moderate self-awareness capabilities and modest self-improving capabilities over time [17].

With regard to consistency in its responses in our study, ChatGPT-3.5 exhibited reasonable short- and medium-term consistency. We think that some variability is actually to be expected, given that LLM chatbots have been developed as AI conversational counterparts and not as medical software tools, of which one would require high repeatability.

Given the currently rapidly expanding literature on the subject, at the time of writing, and to the best of our knowledge, ours was the first study to comparatively assess three of the most widely used LLM chatbots as regards their ability to diagnose challenging ophthalmic case scenarios and to provide advice on further investigation and management. Importantly, we tried to imitate real life in that not all typical clinical symptoms and signs of a condition are always present and to follow the diagnostic thinking process of human and AI in a stepwise fashion rather than present them with a full case description with all the relevant clinical and laboratory/imaging data. In addition, we are the first to evaluate ChatGPT's consistency of responses on the same day and over a period of a few weeks in the above context.

On the other hand, we need to acknowledge our subjectivity in evaluating the chatbots’ responses as well as the fact that we used a limited number of case scenarios, which, despite touching on various ophthalmic subspecialties, may not accurately capture the complexity of other ophthalmic clinical entities. Therefore, we cannot generalize our findings to all eye conditions. Moreover, we used the ChatGPT-3.5 version which, although free to use, is inferior to the more advanced, but requiring subscription fees, GPT-4 version. However, Bing AI chatbot benefits from incorporating GPT-4 in its responses, therefore we may have indirectly involved it as well in our study. Finally, we should keep in mind the time-sensitive nature of our findings, given the constant updates and improvement of LLM chatbots. Future studies could thus indicate even better diagnostic performance of chatbots and provide insight into their consistency in the longer term.

## Conclusions

ChatGPT-3.5 and Bing Copilot chatbots proved to be useful assisting tools in diagnosing and managing certain challenging ophthalmic cases. They both outperformed Google Gemini, although they were inferior to a fellowship-trained ophthalmic specialist. ChatGPT provided fairly consistent responses in the short and medium term. Our findings underscore the potential of LLM chatbots to assist in the diagnostic thinking process; however, they cannot and should not substitute the clinician.
